# Combining Patulin with Cadmium Induces Enhanced Hepatotoxicity and Nephrotoxicity In Vitro and In Vivo

**DOI:** 10.3390/toxins13030221

**Published:** 2021-03-18

**Authors:** Jinling Cui, Shutao Yin, Chong Zhao, Lihong Fan, Hongbo Hu

**Affiliations:** 1 Beijing Advanced Innovation Center for Food Nutrition and Human Health, College of Food Science and Nutritional Engineering, Beijing Key Laboratory for Food Non-Thermal Processing, China Agricultural University, No.17 Qinghua East Road, Haidian District, Beijing 100083, China; cuijinling0420@cau.edu.cn (J.C.); yinshutao@cau.edu.cn (S.Y.); zhaoch0206@cau.edu.cn (C.Z.); 2 College of Veterinary Medicine, China Agricultural University, No.2 Yunamingyuan West Road, Haidian District, Beijing 100193, China

**Keywords:** patulin, cadmium, synergistic toxicity, oxidative stress, JNK1, p53

## Abstract

Food can be contaminated by various types of contaminants such as mycotoxins and toxic heavy metals. Therefore, it is very likely that simultaneous intake of more than one type of food contaminant by consumers may take place, which provides a strong rationale for investigating the combined toxicities of these food contaminants. Patulin is one of the most common food-borne mycotoxins, whereas cadmium is a representative of toxic heavy metals found in food. The liver and kidneys are the main target organ sites for both patulin and cadmium. We hypothesized that simultaneous exposure to patulin and cadmium could produce synergistic hepatotoxicity and nephrotoxicity. Alpha mouse liver 12 (AML12) and Human embryonic kidney (HEK) 293 (HEK293) cell lines together with a mouse model were used to explore the combination effect and mechanism. The results demonstrated, for the first time, that the co-exposure of liver or renal cells to patulin and cadmium caused synergistic cytotoxicity in vitro and enhanced liver toxicity in vivo. The synergistic toxicity caused by the co-administration of patulin and cadmium was attributed to the boosted reactive oxygen species (ROS) generation. c-Jun N-terminal kinase 1 (JNK1) and p53 as downstream mediators of oxidative stress contributed to the synergistic toxicity by co-exposure of patulin and cadmium, while p53/JNK1 activation promoted the second-round ROS production through a positive feedback loop. The findings of the present study extend the toxicological knowledge about patulin and cadmium, which could be beneficial to more precisely perform risk assessments on these food contaminants.

## 1. Introduction

Food can be contaminated by various types of contaminants such as mycotoxins, toxic heavy metals, pesticides, veterinary drugs and illegal food additives. Therefore, it is very likely that simultaneous intake of more than one type of food contaminant by consumers may take place, which provides a strong rationale for investigating the combined toxicities of these food contaminants.

Patulin (PAT), which belongs to fungal secondary metabolites (mycotoxins), is a common contaminant of moldy fruits and products based on them [1]. Previous studies have demonstrated that patulin could produce a diverse range of adverse effects on animal and human health, including immunotoxicity, neurotoxicity, hepatotoxicity and nephrotoxicity [2]. Electrophilic patulin forming covalent adducts with glutathione (GSH) results in the generation of ROS, which is crucial to the induction of organ toxicity [3]. In addition, a number of signaling pathways such as mitogen-activated protein kinase (MAPK), p53, v-akt murine thymoma viral oncogene homolog (AKT) and endoplasmic reticulum stress have been identified to be involved in the toxicity of patulin [3,4,5].

Toxic heavy metals are another major type of food contaminants. Cadmium is one of the most common toxic heavy metals found in food. The toxicities of cadmium mainly include hepatotoxicity, nephrotoxicity, carcinogenicity, teratogenicity and reproductive toxicity [6]. Apoptosis induction in the target organs is considered to be a key event at the cellular level. Oxidative stress induction and MAPK activation have been suggested to contribute to cadmium-induced apoptosis [7,8].

As mentioned above, patulin and cadmium are often found in fruits and rice, respectively; therefore, it is possible that consumers are co-exposed to patulin and cadmium when the meal they eat contains patulin-contaminated fruits and cadmium-contaminated rice. The liver and kidneys are the main target organ sites for both patulin and cadmium. We hypothesized that simultaneous exposure to patulin and cadmium could produce synergistic hepatotoxicity and nephrotoxicity. In our study, this hypothesis was tested using both cell and mouse models.

## 2. Results

### 2.1. Combining PAT and CdCl_2_ Causes a Synergistic Apoptosis Induction In Vitro

**Figure 1:**
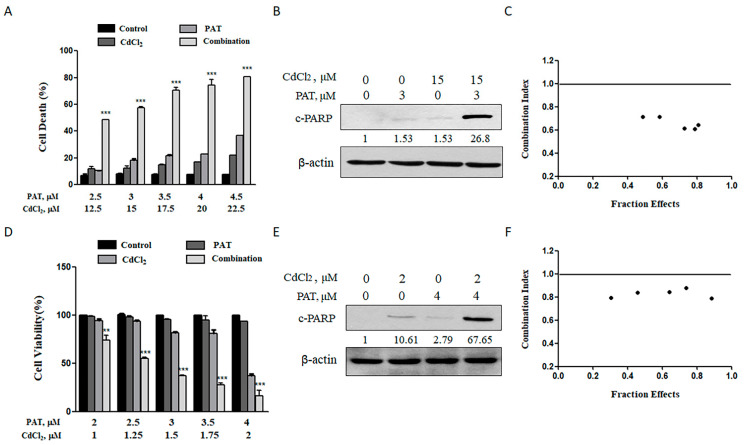
Synergistic effect in cell death can be induced by patulin (PAT) and cadmium chloride (CdCl_2_) in AML12 and HEK293 cells. (**A**) Cell death caused by PAT with or without CdCl_2_ in AML12 cells. Cells were incubated with PAT, CdCl_2_ or both co-administered in a fixed ratio (1:5) for 24 h and cell death was measured. (**B**) Western blot showing the expression of c-PARP in AML12 cells. (**C**) The combination index (CI) was calculated using CompuSyn software. (**D**) Cell death caused by PAT with or without CdCl_2_ in HEK293 cells. Cells were incubated with PAT, CdCl_2_ or both co-administered in a fixed ratio (2:1) for 24 h and the cell number was measured. (**E**) Western blot showing the expression of c-PARP in HEK293 cells incubated with PAT and/or CdCl_2._ (**F**) The combination index (CI) was calculated using CompuSyn software. *n* = 3, ** *P* < 0.01 and *** *P* < 0.001.

For the purpose of assessing the combined toxicity of PAT and cadmium chloride (CdCl_2_), AML12 mouse liver cells and HEK293 human kidney cells were employed to examine the combined hepatocyte and nephrocyte toxicity, respectively. According to our preliminary dose range finding experiments, we chose dose levels of 2.5 to 4.5 μmol/L PAT and 12.5 to 22.5 μmol/L CdCl_2_, which, by themselves, caused minimal or modest apoptosis in AML12 cells. As shown in Figure 1A, treatment with PAT or CdCl_2_ individually induced a slight or modest, but dose-dependent, increase in apoptosis. However, co-treatment caused a significantly higher increase in cell death induction and cleavage of poly(ADP-ribose) polymerase (c-PARP) (Figure 1B). We wondered if the enhanced cell death was a synergistic action. Therefore, the data that the doses of each compound tested covered a range of toxicity from about 30% to 90% were processed to calculate the combination index (*CI*) using CompuSyn software (see Figure S1 in Supplementary Materials). The values of *CI* were lower than 1 for all combinations tested, suggesting that a synergistic effect did exist on AML12 cells by co-treatment with PAT/CdCl_2_ (Figure 1C). For the combined nephrocyte toxicity, the dose levels of 2 to 4 μmol/L PAT and 1 to 2 μmol/L CdCl_2_ were chosen in HEK293 cells based on the same criteria used for hepatocyte toxicity. As shown in Figure 1D–F and Figure S2 in Supplementary Materials, similar results were found in HEK293 cells. Together, these results indicate that combination of patulin and cadmium induced a synergistic hepatocyte and nephrocyte toxicity in vitro.

### 2.2. Synergistic Cell Death Caused by PAT and CdCl_2_ Is Mitochondria-Mediated Caspase-Dependent Apoptosis

**Figure 2:**
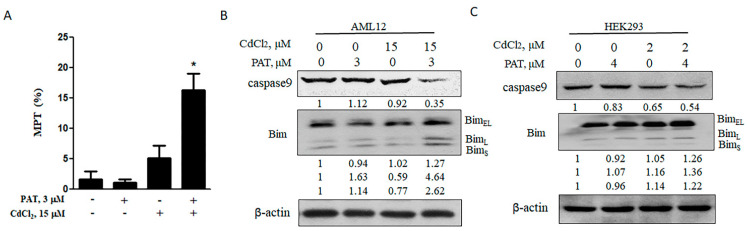
Synergistic cell death induced by PAT and CdCl_2_ is mitochondria-mediated caspase-dependent apoptosis. (**A**) Effect of PAT/CdCl_2_ co-administration on mitochondrial permeability transition (MPT) in AML12 cells. The cells were treated with PAT, CdCl_2_ or their combination for 12 h and the MPT was detected by tetraethyl benzimidazolyl carbocyanine iodide (JC-1) staining. (**B**) Effect of PAT/CdCl_2_ co-administration on mitochondria-mediated apoptosis. AML12 cells were incubated with PAT with or without CdCl_2_ for 24 h, and then, Bim and caspase-9 were assessed. (**C**) Effect of PAT/CdCl_2_ co-administration on mitochondria-mediated apoptosis. HEK293 cells were incubated with PAT with or without CdCl_2_ for 24 h, and then, Bim and caspase-9 were assessed. *n* = 3, * *P* < 0.05.

It was demonstrated that the mitochondrial pathway was activated in response to either patulin or cadmium [9,10]. To determine the contribution of the mitochondrial pathway to the synergistic cell death mediated by PAT and CdCl_2_, we measured the levels of cell mitochondrial membrane potential (MMP) by treatment with each agent alone or their combination. The co-treatment caused a significant decrease in MMP, while the level was not significantly altered by either agent alone (Figure 2A). MMP is tightly regulated by b-cell lymphoma-2 (Bcl-2) family proteins. As expected, Bcl-2 interacting mediator of cell death (Bim), a pro-apoptotic Bcl-2 family protein, was upregulated by the combination of PAT and CdCl_2_, followed by an obvious activation of caspase-9 (Figure 2B,C). These results suggest that the synergistic cell death induced by PAT and CdCl_2_ is mitochondria-mediated caspase-dependent apoptosis.

### 2.3. Enhanced ROS Generation Plays a Critical Role in the Cell Death Caused by Co-Treatment with PAT and CdCl_2_

**Figure 3:**
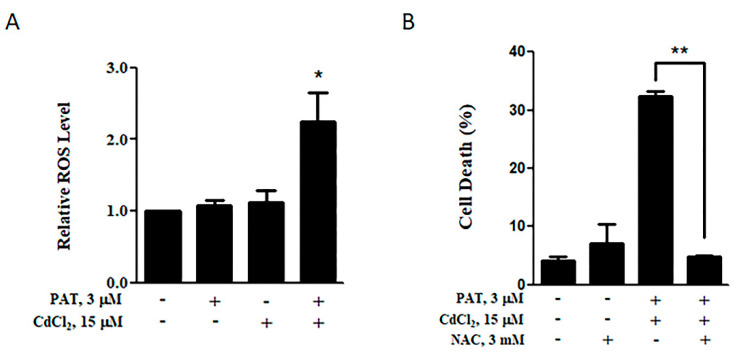
Increased ROS level induced by the co-administration of PAT and CdCl_2_ in AML12 cells. (**A**) Effect of PAT/CdCl_2_ co-administration on ROS level. Cells were incubated with PAT with or without CdCl_2_ for 12 h, and ROS levels were detected using 2′,7′-Dichlorodihydrofluorescein diacetate (DCFH-DA). (**B**) Effect of Nacetyl-1-cysteine (NAC) on PAT/CdCl_2_-induced cell death. Cells were incubated with a combination of PAT and CdCl_2_ or the combination plus NAC for 24 h, and then, cell death was measured. *n* = 3, * *P* < 0.05 and ** *P* < 0.01.

It has been shown that ROS generation plays a key role in the cytotoxic effect induced by either patulin or cadmium [5,7]. We thus evaluated whether the co-treatment with PAT and CdCl_2_ induced a synergistic effect on the level of ROS production. As shown in Figure 3A, combination of PAT and CdCl_2_ induced a significant elevation of ROS compared with PAT or CdCl_2_ treatment alone. To investigate the importance of increased ROS levels in the synergistic toxicity, we measured the influence of the ROS inhibitor Nacetyl-1-cysteine (NAC) on the cell death induction. As expected, treatment with NAC dramatically decreased the synergistic cell death caused by the co-administration (Figure 3B). These results demonstrate that the co-administration of patulin and cadmium boosted ROS generation, which, in turn, led to the synergistic cytotoxicity.

### 2.4. p53 Contributes to the Synergistic Effect Induced by the Combination of PAT and CdCl_2_

**Figure 4:**
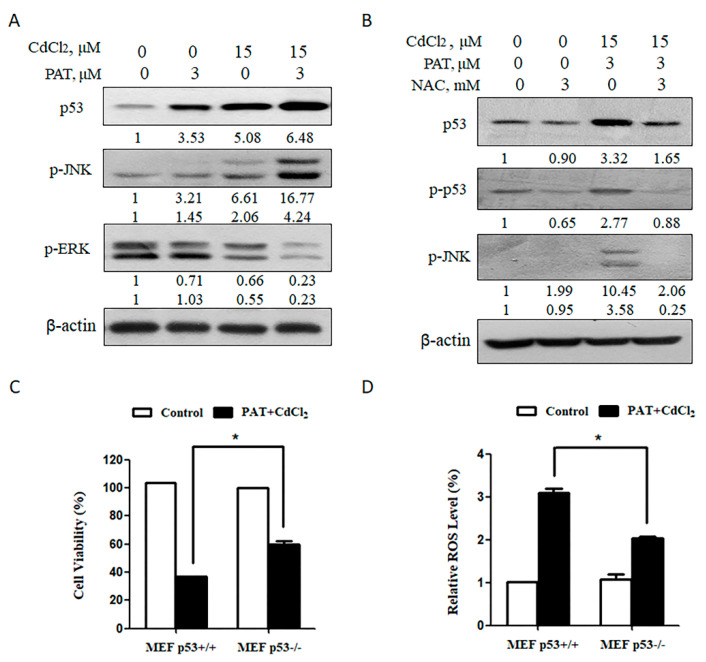
p53 contributes to the synergistic effect induced by the combination of PAT and CdCl_2_. (**A**) Effect of the combination of PAT and CdCl_2_ on p53 and MAPK pathway. Western blot showing the expression of p53, p-JNK and p-ERK in AML12 and HEK293 cells treated with PAT and/or CdCl_2._ (**B**) Effect of NAC on PAT/CdCl_2_-induced activation of p53 and JNK. AML12 cells were incubated with the combination of PAT and CdCl_2_ or the combination plus NAC for 24 h, and then, p53, p-p53 and p-JNK were assessed. (**C**) Co-treatment of PAT and CdCl_2_ induced growth inhibition in p53+/+ or p53-/- MEF cells. The cells were incubated with PAT and CdCl_2_ for 24 h, and cell viability was measured. (**D**) ROS levels were detected in p53 knockout/wild-type MEF cells by co-treatment of PAT and CdCl_2_. The cells were incubated with PAT and CdCl_2_ for 12 h; then, the ROS levels were detected by using DCFH-DA. *n* = 3, * *P* < 0.05.

It was shown that activated p53 was involved in patulin- or cadmium-mediated toxicity [4,11,12]; therefore, we wondered if p53 was involved in the synergistic cytotoxicity induced by PAT and CdCl_2_. The results showed that co-treatment with PAT and CdCl_2_ increased the expression of p53 and p-JNK but reduced phosphor-extracellular regulated protein kinase (p-ERK) (Figure 4A). As shown in Figure 4B, pretreatment with NAC dramatically decreased the phosphorylation of p53 and JNK induced by the combination. Our previous study showed that p53 plays a pro-oxidant role by patulin treatment [4]. We next investigated the contribution of p53 to the enhanced oxidative stress by combining patulin with cadmium. Cell death induction was decreased in p53-/- mouse embryonic fibroblast (MEF) cells in comparison with that in p53+/+ MEF cells (Figure 4C). Consistent with the reduction in apoptosis, a decreased ROS level was detected in p53-/-MEF cells than that found in p53+/+ MEF cells (Figure 4D). The results indicate that p53 activation exerts a pro-oxidant function that leads to ROS generation by co-administration of PAT and CdCl_2_.

### 2.5. JNK1 Activation Contributes to the Synergistic Effect Caused by the Combination of PAT and CdCl_2_

**Figure 5:**
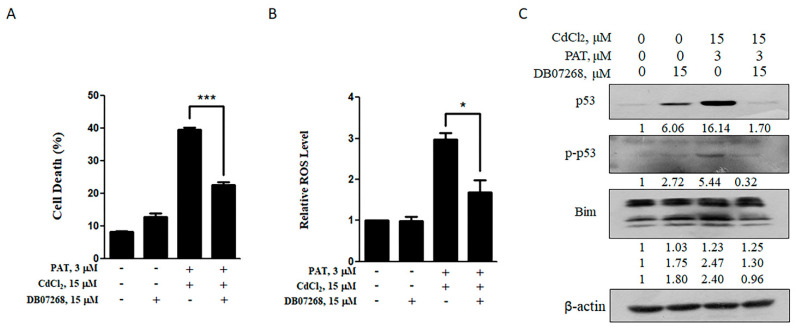
JNK1 inhibitor (DB07268) can rescue the synergistic effect caused by co-treatment with PAT and CdCl_2_. (**A**) Effect of DB07268 on PAT/CdCl_2_-induced cell death. The cells were treated with the combination of PAT and CdCl_2_ or the combination plus DB07268 for 24 h, and then, cell death was measured. (**B**) Effect of DB07268 on PAT/CdCl_2_-induced ROS generation. The cells were incubated with the combination of PAT and CdCl_2_ or the combination plus DB07268 for 12 h, and ROS levels were detected by using DCFH-DA. (**C**) Effect of DB07268 on activation of p53 and Bim induced by PAT and CdCl_2_. AML12 cells were incubated with the combination of PAT and CdCl_2_ or the combination plus DB07268 for 24 h, and then, p53, p-p53 and Bim were measured by Western blotting. *n* = 3, * *P* < 0.05 and *** *P* < 0.001.

Having found p-JNK induction obviously by PAT/CdCl_2_ combination, we used a JNK inhibitor to see if it protected against cell death as JNK exerted its pro-apoptotic function generally. As expected, the JNK1 inhibitor DB07268 reduced the combination-induced cell death significantly (Figure 5A). Consistent with the reduction in apoptosis, a significantly decreased ROS level was detected by pretreatment with DB07268 (Figure 5B). Meanwhile, the activation of p53 and Bim induced by the combination was nearly reduced to control level (Figure 5C). These results suggest a crucial role for JNK1 in the synergetic cell death induced by PAT and CdCl_2_.

### 2.6. Combination of PAT and CdCl_2_ Caused Hepatic and Renal Injury In Vivo

**Figure 6:**
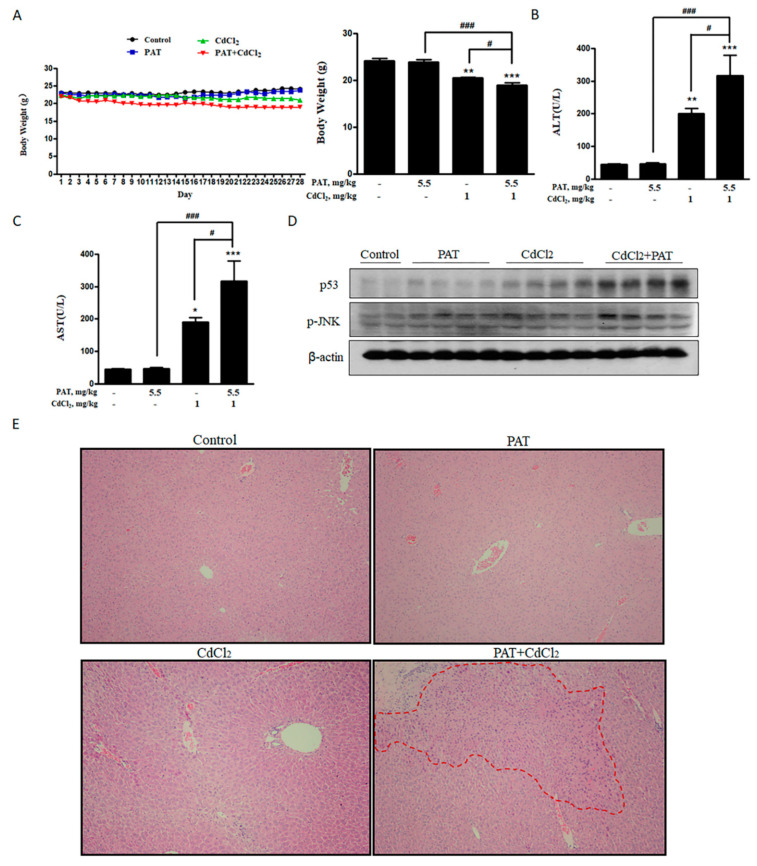

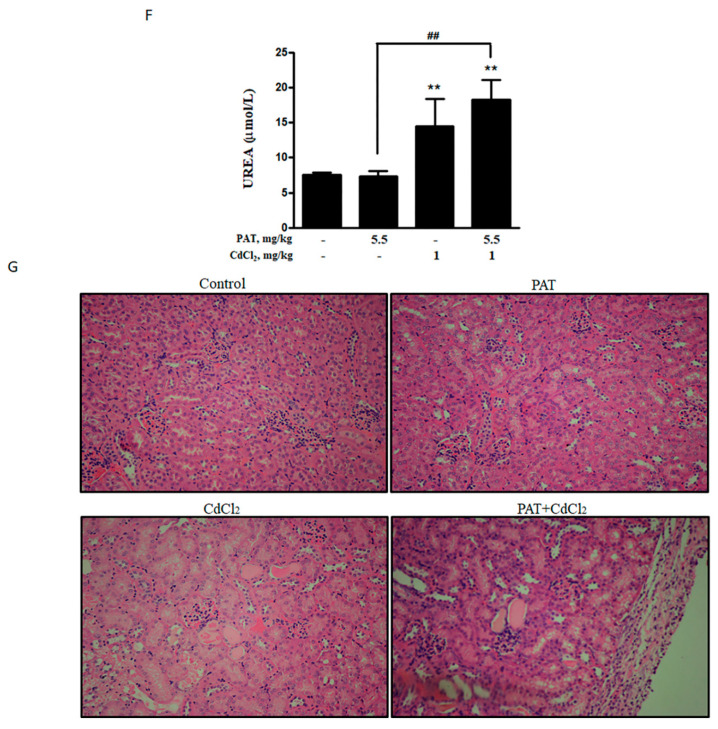
Combination of PAT and CdCl_2_ causes liver and kidney damage in vivo. (**A**) Body weight of mice. (**B**,**C**) Effects of PAT and/or CdCl_2_ on serum alanine transaminase (ALT) and aspartate transaminase (AST). (**D**) Western blot showing the effects of PAT and/or CdCl_2_ on p53 and p-JNK of liver tissues. (**E**) Liver pathological damage caused by PAT and/or CdCl_2_ combination as measured by hematoxylin–eosin (H&E) staining of sections of liver tissues. (**F**) Effects of PAT and/or CdCl_2_ on serum urea. (**G**) Kidney pathological injury caused by PAT and/or CdCl_2_ as assessed by H&E staining of sections of kidney tissues. *n* = 5, “*” represents the significance compared with control group, where * *P* < 0.05, ** *P* < 0.01 and *** *P* < 0.001. “^#^” represents the significance compared between cotreatment of PAT and CdCl_2_ with PAT or CdCl_2_ treatment alone. ^#^ *P* < 0.05, ^##^ *P* < 0.01 and ^###^ *P* < 0.001.

After investigating the toxicological mechanism of co-administration of PAT and CdCl_2_ in vitro, we further validated these findings in vivo. The combination of PAT and CdCl_2_ decreased the body weight of mice compared with PAT or CdCl_2_ treatment alone (Figure 6A). Biochemical parameters of liver damage, serum alanine transaminase (ALT) and aspartate transaminase (AST) were significantly increased by the co-administration (Figure 6B,C). Acidophilic change was only found in PAT/ CdCl_2_ combination (Figure 6E), suggesting a severe liver injury compared with PAT or CdCl_2_ treatment alone. The key kidney damage marker, serum urea, was increased significantly, suggesting a severe kidney injury induced by co-treatment with PAT and CdCl_2_ (Figure 6F). Hematoxylin–eosin (H&E) staining showed that treatment with CdCl_2_ resulted in mild tubule swelling and protein casts. The co-administration group showed serious tubule swelling, protein casts and infiltrate of neutrophil polymorphs (Figure 6G). Consistent with the change in protein expression in vitro, p53 and p-JNK were upregulated following co-administration of PAT and CdCl_2_ in mice (Figure 6D).

## 3. Discussion

Previously, toxicological evaluation of food contaminants was generally performed under a single exposure setting. Recently, the combined toxic effect of food contaminants has drawn increasing attention due to the fact that consumers are often exposed not to a single contaminant but to a combination of contaminants. However, most of the studies have focused on the interaction among same the class of food contaminants such as the combined toxicity of mycotoxins [13,14] or the combined toxicity of heavy metals [15,16]. Due to the co-occurrence of mycotoxins in food, co-exposure to mycotoxins was considered a severe issue. Studies have elucidated the toxicological interactions between them. Patulin and ochratoxin A induced a synergistic effect at low doses but antagonistic action at higher levels [13]. The toxicity, higher than additive toxicity, was induced by citrinin and patulin when combined with ochratoxin A or ochratoxin B in Lilly Laboratories cell porcine kidney 1 (LLC-PK1) cells [17]. As, Cd and Pb induced synergistic action in glial and neuronal functions [18]. The combination of cadmium and molybdenum caused synergistic toxicity through mitochondria-mediated oxidative stress and apoptosis [19]. However, data on the combined toxicity of different types of food contaminants are rarely available. Given the possibility that consumers are often exposed to multiple types of contaminants, investigations on the toxicological interactions among different types of food contaminants are clearly needed, especially for contaminants that share the same toxic target organs. Patulin is one of the most common food-borne mycotoxins, whereas cadmium is a representative of toxic heavy metals found in food. The toxicities of single exposure have been well documented, and hepatotoxicity and nephrotoxicity are the major adverse effects of either patulin or cadmium [2,6,20]. The present study moved from single exposure to combined exposure, and the results demonstrated, for the first time, that the co-exposure of liver or renal cells to patulin and cadmium caused synergistic cytotoxicity in vitro and enhanced hepatotoxicity in vivo. The findings of our results extend the toxicological knowledge about patulin or cadmium, which could be beneficial to more precisely perform risk assessment on these food contaminants.

It was reported that patulin has a strong affinity for SH groups due to its electrophilic attribute [21,22], which was proven by the research showing that patulin induced a significant decrease in GSH activity by forming an adduct with SH groups [23]. In addition, a disrupted cellular GSH system contributes to ROS formation, which is suggested to play a critical role in cadmium-induced toxicity [24]. We speculated that the synergistic cytotoxicity caused by co-administration of patulin and cadmium was probably attributed to augmented ROS generation. Indeed, the results showed that a significantly elevated ROS level was induced in response to the co-occurrence in comparison with exposure to each agent alone, whereas suppression of ROS by an antioxidant abolished the combination-induced cell death. The data clearly support our hypothesis. To decipher the downstream signaling pathway that contributed to the combination-induced ROS-mediated cytotoxicity, a number of apoptosis-related molecules were analyzed, and the results demonstrated that an enhanced phosphorylation of p53 and JNK was detected in the combination-treated cells compared with that found in the treatment with either agent alone. Inhibition of ROS led to abolishment of the activation of p53 and JNK, while inhibiting JNK1 activation resulted in inactivating p53. The functional role of p53 or JNK was further validated by p53 knockout/wild-type MEF cell system or JNK1 inhibitor. In addition, inhibiting either JNK1 or p53 caused significant attenuation of the combination-induced ROS generation. These results together indicate that increased activation of the ROS-JNK1-p53 axis contributed to the synergistic toxicity caused by co-administration of patulin and cadmium, while p53/JNK1 activation promoted the second-round ROS generation through a positive feedback loop. In our finding, the combination of patulin and cadmium activated Bim, with obvious increased L and S subunits but little change to the EL subunit. As demonstrated in a previous study, L and S subunits of Bim are more potent inducers of cell death than the EL subunit [25]. We also found that ERK phosphorylation was decreased by the combination. ERK has been reported to mediate the phosphorylation and degradation of Bim [26]. The decrease in p-ERK might be one of the reasons for Bim upregulation by co-administration of patulin and cadmium. Collectively, the data provided a mechanistic explanation for the synergistically toxic interaction between patulin and cadmium.

## 4. Conclusions

In summary, the present study demonstrated that co-administration of patulin and cadmium triggered a strong synergistic toxic consequence through increased oxidative stress. JNK1 and p53 as downstream mediators of oxidative stress contributed to the synergistic toxicity by co-exposure of patulin and cadmium. The findings implied that the combined toxicity should be taken into account when performing risk assessments for the presence of patulin and cadmium in food.

## 5. Materials and Methods

### 5.1. Chemicals and Reagents

Patulin (PAT), cadmium chloride (CdCl_2_), Nacetyl-1-cysteine (NAC) and 2′,7′-Dichlorodihydrofluorescein diacetate (DCFH-DA) were purchased from Sigma-Aldrich (St. Louis, MO, USA.). DB07268 was purchased from Med Chem Express (Danvers, MA, USA.). Antibodies specific for cleaved PARP (9532), p53(2524), p-p53(9284), p-JNK (9251), p-ERK (9101), caspase-9 (9508), Bim (2933) and β-actin were purchased from Cell Signaling Technology (Beverly, MA, USA.). PAT (10 mg) was dissolved in 1.3 mL ultrapure water to make it a 50-mM solution. PAT (2 mM) was obtained by mixing 100 μL PAT (10 mM) with 400 μL ultrapure water. CdCl_2_ (18 mg) was dissolved in 1.23 mL ultrapure water to make it a 80-mM solution. CdCl_2_ (10 mM) was obtained by mixing 100 μL CdCl_2_ (80 mM) with 700 μL ultrapure water. CdCl_2_ (1 mM) was obtained by mixing 50 μL CdCl_2_ (10 mM) with 450 μL ultrapure water.

### 5.2. Cell Culture and Treatments

AML12 cells were obtained from the American Type Culture Collection (ATCC), which were cultured in Dulbecco’s Modified Eagle Medium (DMEM)/F12 medium (HyClone, Logan, UT, USA) with 1% insulin transferrin selenium (ITS) and supplemented with 10% fetal bovine serum. HEK293 and MEF cells were obtained from ATCC, which were cultured in Dulbecco’s Modified Eagle Medium (DMEM) (HyClone, Logan, UT, USA) supplemented with 10% fetal bovine serum. After plating for 24 h, the cultured cells were grown to 50% confluence. Then, the medium was aspirated, and the treatment with different agents was given.

### 5.3. Apoptosis Evaluation

Treated cells were analyzed by flow cytometry after staining with Annexin V and PI (MBL International Corporation, Boston, MA, USA) for 30 min. The second method was immunoblot analysis of PARP cleavage.

### 5.4. Crystal Violet Staining

After treatment, the medium was removed and 1% glutaraldehyde solution was added to fix the cells for 15 min. After washing with phosphate buffer saline (PBS), the cells were stained with a 0.02% crystal violet solution for 30 min. After air-drying, 70% ethanol was added for solubilization. The absorbance at 570 nm was examined using a microplate reader with a 405-nm reference filter.

### 5.5. Western Blotting

Cells were lysed with radio-immuno-precipitation assay (RIPA) buffer containing protease inhibitor. The denatured proteins were loaded onto the gel and assessed by SDS-PAGE electrophoresis. After transferring onto a nitrocellulose membrane, the membrane was incubated with primary antibodies followed by the corresponding secondary antibody. The blots were recorded on X-ray film using enhanced chemiluminescence. The quantification of Western blot can be found in Figure S3.

### 5.6. Measurement of ROS

Cells were incubated with PAT (3 μM) and/or CdCl_2_ (15 μM) for 12 h. Thirty minutes before harvesting the cells, free medium containing DCFH-DA (20 μM) was added. After washing with PBS, the cells were harvested and resuspended. The fluorescent dichlorodihydrofluorescein (DCF) was examined using a flow cytometer.

### 5.7. Measurement of MMP

Cells were incubated with PAT (3 μM) and/or CdCl_2_ (15 μM) for 12 h. Mitochondrial membrane potential (MMP) was analyzed by flow cytometry after staining with JC-1 according to the manufacturer’s instructions (JC-1 kit, Solarbio Life Science, Beijing, China).

### 5.8. Combination Index Calculation

The synergistic effects between PAT and CdCl_2_ were analyzed by calculating the combination index (*CI*) using CompuSyn 2.0 software (CompuSyn, Paramus, NJ, USA). The cells were incubated with various concentrations of PAT, CdCl_2_ and their combination, and the total inhibitory effect was assessed by cell viability or cell death (see Figures S1 and S2 in Supplementary Materials). *CI* < 1, *CI* = 1 and *CI* > 1 indicate synergism, additive effect and antagonism, respectively.

### 5.9. Animals and Treatments

Male C57BL/6N mice weighing 20.5 ± 1.0 g were purchased from Vital River (Beijing, China) and were employed in the present study. The mice received a commercial standard mouse cube diet (Beijing Keaoxieli Feed Company, China). After acclimatization, the mice were randomly divided into 4 groups (*n* = 5): Control group, PAT group (5.5 mg/kg/i.g.), CdCl_2_ group (1 mg/kg/i.p.) and combination group. The mice were administrated with agents every day for 27 d continuously. Male mice and the doses were chosen according to previous toxicological studies of PAT or CdCl_2_.The mice were sacrificed 24 h after the last administration for the collection of plasma, liver and kidney tissues. Sera received from centrifugal plasma were stored at −80 °C. Except for fixing in neutral buffered formalin for H&E staining, liver and kidney tissues were stored at −80 °C for Western blotting.

### 5.10. Biochemical Assay

Serum levels of ALT, AST and urea were measured according to the manufacturer’s instructions (Nanjing Jiancheng Institute of Biotechnology, Nanjing, China).

### 5.11. Histopathology

Fixed liver and kidney tissues were processed to 5-μm thick paraffin sections and stained with hematoxylin and eosin (H&E).

### 5.12. Statistical Analysis

The results were obtained three times and data were presented as mean ± standard deviation (SD). The data were evaluated using a one-way ANOVA followed by Tukey’s post-hoc test. *P* < 0.05 was considered statistically significant.

## Data Availability

Data is contained within the article or supplementary material.

## Supplementary Materials

The following are available online at https://www.mdpi.com/2072-6651/13/3/221/s1, Figure S1: CompuSyn report—AML12, Figure S2: CompuSyn report—HEK293, Figure S3: Quantification of Western blot.
