# Supplementary figures and images for: Combining Patulin with Cadmium Induces Enhanced Hepatotoxicity and Nephrotoxicity In Vitro and In Vivo
Source: Toxins (Basel). 2021 Mar 18;13(3):221. doi: 10.3390/toxins13030221 (PMC8003173; doi:10.3390/toxins13030221)

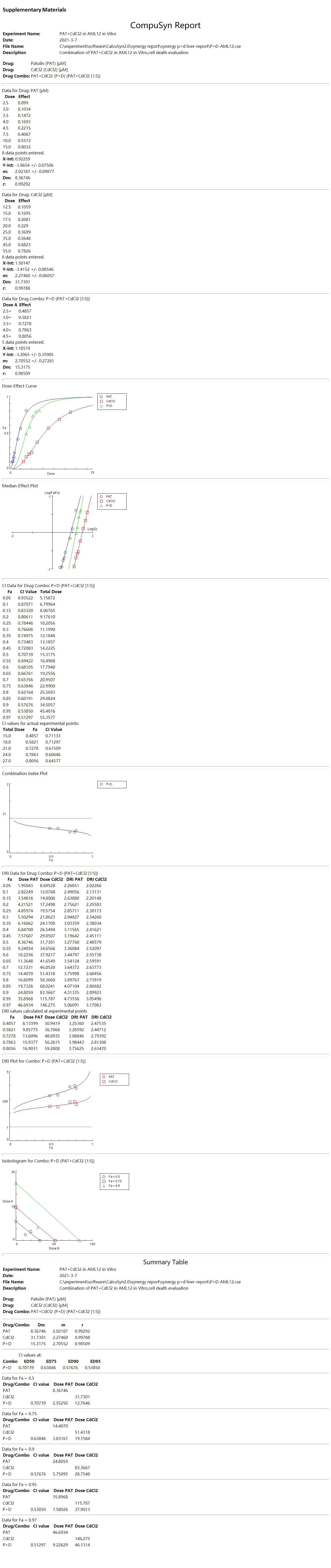

Supplement: Supplementary file 1 [file toxins-13-00221-s001.zip › Supplementary Materials/Figure S1-CompuSyn Report-AML12.jpg]

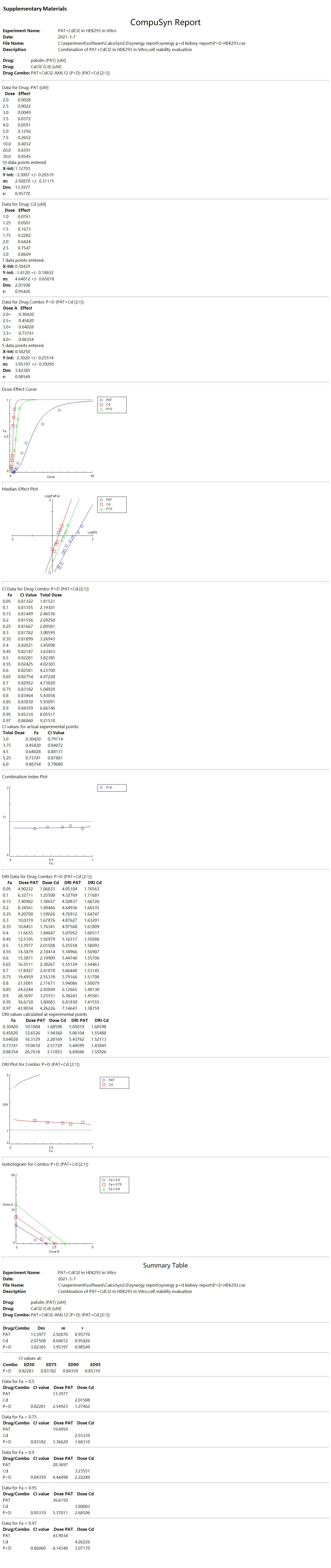

Supplement: Supplementary file 1 [file toxins-13-00221-s001.zip › Supplementary Materials/Figure S2-CompuSyn Report-HEK293.jpg]

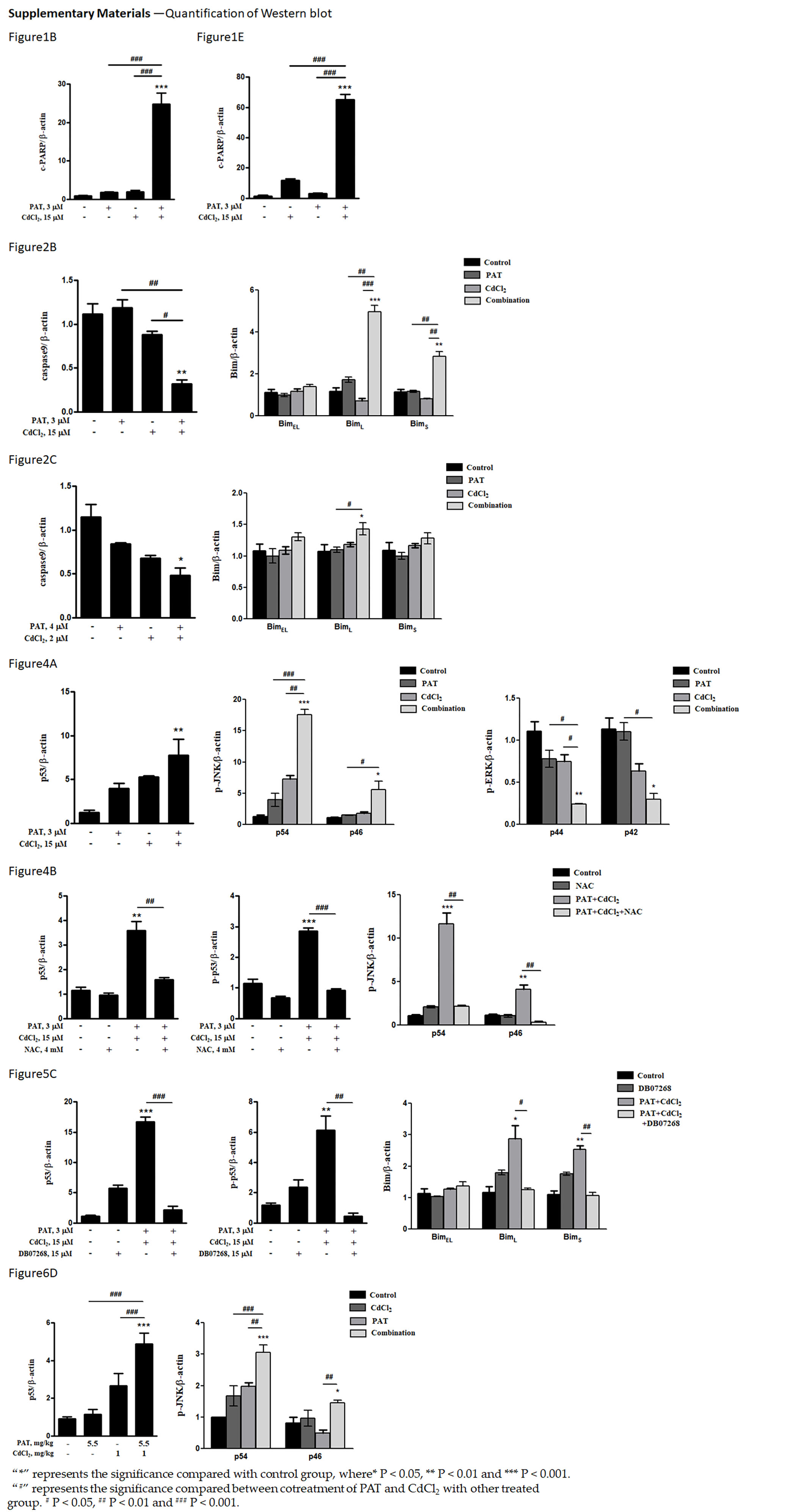

Supplement: Supplementary file 1 [file toxins-13-00221-s001.zip › Supplementary Materials/Figure S3-Quantification of Western blot .jpg]
